# The Influence of Electroconvulsive Therapy (ECT) on Brain-Derived Neurotrophic Factor (BDNF) Plasma Level in Patients with Schizophrenia—A Systematic Review and Meta-Analysis

**DOI:** 10.3390/jcm12175728

**Published:** 2023-09-02

**Authors:** Anna Maria Szota, Beata Kowalewska, Małgorzata Ćwiklińska-Jurkowska, Wiktor Dróżdż

**Affiliations:** 1 Department of Psychiatry, Ludwig Rydygier Collegium Medicum in Bydgoszcz, Nicolaus Copernicus University in Toruń, Curie-Skłodowskiej Street 9, 85-094 Bydgoszcz, Poland; beata.kowalewska@cm.umk.pl (B.K.); wikdr@cm.umk.pl (W.D.); 2 Department of Biostatistics and Biomedical Systems Theory, Ludwig Rydygier Collegium Medicum in Bydgoszcz, Nicolaus Copernicus University in Toruń, Jagiellońska Street 13-15, 85-067 Bydgoszcz, Poland; mjurkowska@cm.umk.pl

**Keywords:** schizophrenia, meta-analysis, electroconvulsive therapy (ECT), brain-derived neurotrophic factor (BDNF)

## Abstract

The main aim of this systematic review and meta-analysis is to establish whether there is a correlation between the brain-derived neurotrophic factor (BDNF) level and electroconvulsive therapy (ECT) treatment and the reduction in psychotic symptoms in patients diagnosed with schizophrenia. A systematic search of PubMed/Medline, Cochrane Library, Web of Science, Scopus and Embase was conducted up to March 2023. Inclusion criteria: studies in which adult patients with schizophrenia treated with antipsychotic medication received ECT therapy and had the BDNF level measured before and after ECT treatment. Exclusion criteria: animal and in vitro studies or studies not involving complete information about the treatment and concentration of BDNF in plasma. The risk of bias was assessed using Egger’s regression-based test for meta-analysis with continuous outcomes. Six studies comprising 248 individuals with schizophrenia were included. A statistically significant increase in BDNF levels after ECT treatment was observed only in two studies (*p* < 0.001 and *p* < 0.027, respectively), whereas in four other studies, an upward trend without statistical significance was noticed. The estimated overall size effect revealed that ECT therapy caused a slight change in the BDNF level but without statistical significance (ES = −0.328). Different numbers of ECT procedures (4-10), final measurement of the BDNF level made at a different time point, using bilateral or unilateral electrode positioning during ECT and treatment with different combinations of typical or atypical antipsychotic medications may be potential reasons for the lack of statistical significance in the changes in BDNF levels after treatment. Data regarding the measurement of BDNF levels pre and post ECT therapy in patients with schizophrenia are very limited without an extended follow-up period and evaluation of mental health change. Our meta-analysis showed that treatment with ECT therapy and antipsychotic medication increases serum BDNF levels in patients with drug-resistant schizophrenia compared to patients treated with medication only; however, this effect is not statistically significant.

## 1. Introduction

Schizophrenia is a neuropsychiatric disorder that is characterized by deficits in thought processing, emotional responsiveness and cognition [1,2,3]. The cause of this disabling disorder remains unclear, but a growing body of evidence has pointed to neurodevelopmental processes in which neurotrophic factors are involved [4]. Brain-derived neurotrophic factor (BDNF) is an important neurotropin and can be found both in the human brain (cortex, hippocampus and forebrain) and peripheral blood [5]. BDNF plays a significant role in neurodevelopment and the survival, function and repair of neurons, including dopaminergic and serotoninergic neurons [5,6]. Also, BDNF may be an essential modulator of cognitive functions like memory, learning and thinking. Moreover, BDNF promotes neuronal plasticity in the nervous system by improving the survival, growth and differentiation of the nerve synapses [5,7,8].

Research conducted so far has revealed a reduction in BDNF concentrations both in peripheral blood [9,10,11,12] and in brain tissue (post-mortem studies) in patients with schizophrenia, [12,13] when compared to healthy control subjects. Additionally, the concentration of mRNA BDNF in the brain of schizophrenic patients is significantly decreased in comparison to healthy controls [12,14]. Based on these results, we may conclude that a low level of BDNF is likely to mediate in the neurodevelopmental pathway of schizophrenia by affecting new neuron development, synaptogenesis and neuronal communication. Decreased production of BDNF also correlates with cognitive dysfunctions (problems with memory and learning), neurodegeneration and the intensification of apoptosis observed in schizophrenia. Therefore, the effectiveness of antipsychotic drugs used in schizophrenia treatment or electroconvulsive therapy (ECT) used in drug-resistant schizophrenia should be associated with increased BDNF concentration and the resolution of disease symptoms. However, the results of scientific research are inconsistent in this matter. A number of studies demonstrated that serum BDNF concentrations were significantly increased in patients regularly medicated with antipsychotic drugs regardless of the dose [15,16] and in patients medicated for a long time, at least 10 years [17]. Moreover, Pedrini et al. [18] demonstrated that not only does chronic administration of clozapine increase BDNF concentration, but it is also correlated with clozapine dose. Also, in drug-naive first-episode schizophrenia, BDNF concentration is decreased as administration with antipsychotic drugs normalizes its level [19]. With regard to ECT, a twofold influence on the concentration of BDNF was observed. Li et al. [10] reported an increased concentration of BDNF after ECT and antipsychotic medication whereas Akbas et al. [20] and Fernandez et al. [21] obtained opposite results. Their studies demonstrated that BDNF concentration was increased when only antipsychotic drugs were used, not in combination with ECT therapy. It may suggest that an increased concentration of BDNF is a result of using antipsychotic medication, not ECT itself.

The aim of this systematic review and meta-analysis was to determine whether the use of ECT is associated with a change in the concentration of BDNF in patients diagnosed with treatment-resistant schizophrenia compared to the concentration of this factor in patients treated with antipsychotics only.

## 2. Material and Methods

### 2.1. Data Sources and Search Strategy

This meta-analysis was prepared according to the PRISMA (Preferred Reporting Items for Systematic Reviews and Meta-Analyses) statement guidelines [22]. The protocol was registered on the International Prospective Register of Systematic Reviews (PROSPERO), registration number: CRD 42023417335.

Electronic literature search of PubMed, Medline, Medline Complete, Web of Science, Cochrane Library, Scopus and Embase from the earliest available date to March 2023 was conducted. The following terms/keywords were used: [‘schizophrenia’ or ‘psychotic disorder’], [‘electroconvulsive therapy’ or ‘ECT’] and [Brain-derived Neurotropic Factor or BDNF] were used in various combinations. Additional articles were searched by crosschecking the references and other available sources. Articles were selected by using a two-stage process: title and abstract screening and full-text assessment.

### 2.2. Data Extraction

Titles and abstracts were screened, and full-text articles were further assessed for eligibility in an independent manner (A.S.; B.K.). Data extraction of full-text articles was performed by 2 independent coders (A.S.; B.K.). The selection of the articles was conducted on the basis of the inclusion and exclusion criteria.

### 2.3. Study Inclusion and Exclusion Criteria

The inclusion criteria were as follows: (1) prospective/retrospective case control studies/prospective/retrospective cohort studies; randomized control studies (RCT), (2) participants diagnosed with schizophrenia according to the International Classification of Diseases, tenth edition (ICD-10) or Diagnostic and Statistical Manual of Mental Disorders, third to fifth edition (DSM-3, DSM-4, DSM-4TR, DSM-5), (3) course of ECT with both bilateral and unilateral electrode placements included, (4) studies that reported BDNF concentration before and after treatment with ECT and (5) articles written in English and Russian. There was no restriction on antipsychotic medication type and dosage. Exclusion criteria: (1) in vitro studies, (2) animal studies, (3) literature reviews, (4) conference abstract without complete methodology and (5) articles with data overlapping with those articles that were already included in the meta-analysis.

**Figure 1:**
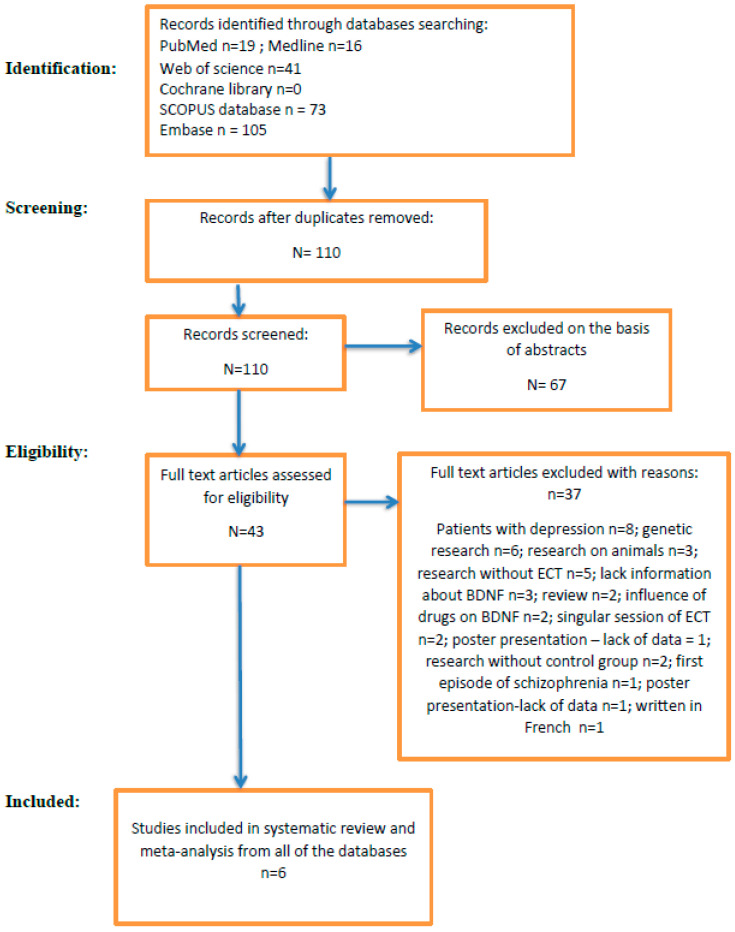
PRISMA 2020 flowchart for systematic reviews. Identification of new studies via databases and registers.

The PRISMA flowchart of the study selection process is presented in Figure 1.

### 2.4. Data Collected

For the estimation of the overall effect size (changes in BDNF before and after ECT), meta-analysis was conducted with continuous outcomes on raw data that are provided on the prepared dataset after PRISMA analysis. Measures of central tendency (averages) and precision measures (as SD) were collected together with sample sizes. Sample averages of variables representing the measures of BDNF (at two time points: first pharmacology treatment and for the same patients with pharmacology treatment additionally after ECT—paired measures) were collected.

### 2.5. Data Synthesis and Statistical Analysis

The studies included in this meta-analysis were from different locations and performed on different populations, so the random-effect model was chosen. The overall effect summarizes well the individual studies if the variances between the different effects are natural, i.e., not large. The study of heterogeneity is proposed to determine to what extent differences between the results obtained by the individual studies affect the overall effect created in the meta-analysis. The heterogeneity was assessed by between-study variance τ^2^. Homogeneity was analyzed by testing whether the variability between studies τ^2^ is equal to zero. For this purpose, Q statistic with *p*-value based on chi-square distribution with k-1 degrees of freedom was applied (where k is the number of studies). Additionally, I^2^ and H^2^ were calculated to assess heterogeneity.

Effect size measures were adjusted by Hedges’ g [23,24]. Hedges’ g, called corrected effect size, is a measure of effect. It is the standardized effect size for the difference between means and uses the sample size weighted pooled standard deviation.

Random-effect weights were estimated by using inverse variance (weights including both within-study and between-study variance). 

The estimation of the effect was performed by using the iterative method calculating the restricted maximum likelihood estimator (REML).

For adjustment of standard error, the truncated Knapp–Hartung [25] method was applied which truncates the value if it is less than 1 when estimating the variance–covariance matrix. Hartung–Knapp method for random-effect meta-analysis gives more adequate error rates than the Der Simonian and Laird method, especially for a few studies [26]. This method is also recommended when the contributed studies’ precisions vary [27].

Publication bias appears for the reason that studies with preferable outcomes are more likely to be published [28]. Consequently, the published results may be biased toward a certain direction. The publication bias analysis was performed by conducting Egger’s regression-based test for meta-analysis with continuous outcomes [29,30].

Egger test for asymmetry is obtained by regressing the standardized effect on the precision (1/SE):

Effect/SE = α + β(1/SE) + ε,

where Effect is the estimated effect, SE is the standard error of Effect, and ε is random noise. The size of α indicates the extent of asymmetry. Egger’s test estimates the statistics based on the *t*-distribution. Test of intercept α = 0 is based on t-distribution with k-2 degrees of freedom. Publication bias was additionally visualized using funnel plots. Funnel plot is visual method that relates the bias to the asymmetry of the funnel plot. On such plots, the studies’ effect sizes against their standard errors are presented.

The trim-and-fill analysis of publication bias [31,32] was also applied for the estimation of the effect size. It is a method of testing and adjusting for publication bias in meta-analysis. The side of imputation for trim-and-fill analysis based on Egger test was chosen.

Meta-analysis was performed with the usage of analytical tool reporting system PS IMAGO Pro 9.0 with the IBM SPSS Statistics 29 analytical engine (https://en.predictivesolutions.pl/ps-imago-pro,en, accessed on 1 August 2023).

## 3. Results

The search of Pub Med, Medline, Web of Science Cochrane Library, Scopus and Embase yielded 254 records. After removing 144 duplicate records and an additional 67 records on the basis of abstract content, 43 records were left for eligibility. After further analysis, only six of them fulfilled inclusion criteria and were included in this systematic review and meta-analysis.

The six included studies involved adult subjects with mean age varying from 32 [20] to 38 [10] years and a proportion of men from 70% [21] to 100% [20]. Each study was conducted in a different country, one in Brazil [21], one in China [10], one in Egipt [33], one in Lithuania [34], one in Russia [35] and one in Turkey [20]. The patients were diagnosed with schizophrenia [10,20], refractory schizophrenia [21] or treatment-resistant schizophrenia [33,34,35]. All patients were treated with different combinations of typical (haloperidol) and atypical (aripiprazole, clozapine, quetiapine, olanzapine, paliperidone, risperidone) drugs [10,20,21,33,34,35]. The ECT courses were conducted with bilateral frontotemporal electrode placement with one exception (unilateral frontotemporal electrode placement) [21]. The number of ECT procedures varied from 3 [35] to 20 [34]. Table 1 shows the characteristics of the included studies.

As reports chosen for this systematic review and meta-analysis come from different locations, the random-effect model was chosen, and consequently, the heterogeneity of the data had to be evaluated. To study the heterogeneity of studies, Q, τ^2^, H^2^ and I^2^ values are given (Tables S1 and S2, attached as supplementary files). The higher the values, the greater the heterogeneity of the study. Analysis of homogeneity does not reject the hypothesis of homogeneity (Table S1 (*p* = 0.066), attached as a supplementary file). Heterogeneity measures given in Table S2, attached as a supplementary file, confirm the lack of considerable heterogeneity. For example, statistic I^2^ = (H^2^ − 1)/H^2^ = 52.29% indicates medium heterogeneity, where H^2^ = Q/(k − 1) = 10.342/5 = 2.096 (Table S2, attached as a supplementary file).

**Figure 2:**
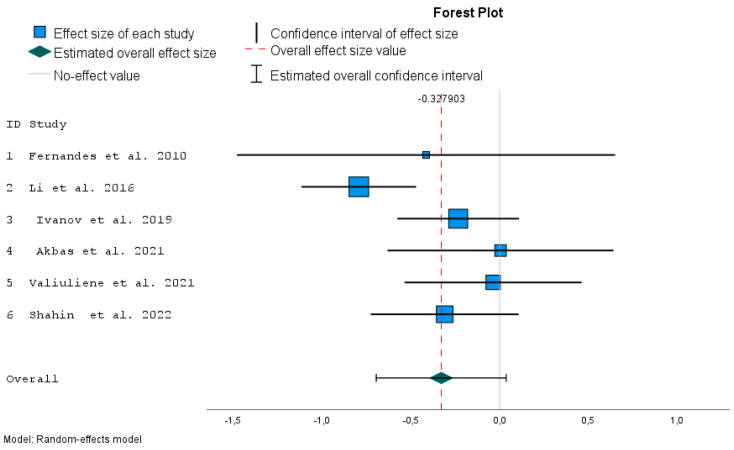
Forest plot for comparison of effect for schizophrenia patients before and after ECT with overall effect size [10,20,21,33,34,35].

The I^2^ estimator indicates the percentage of the observed variance that comes from the true difference in the size of the individual studies’ effects. From a graphical point of view, the forest plot (Figure 2) reflects how much individual confidence intervals overlap.

τ^2^ (tau-squared) equal to 0.059 is the variance of observed effects. Because the variance between study populations is not too large, we have a basis for an overall summary of the effect. Additionally, we might expect a similar result for the common (fixed) effect model.

**Figure 3:**
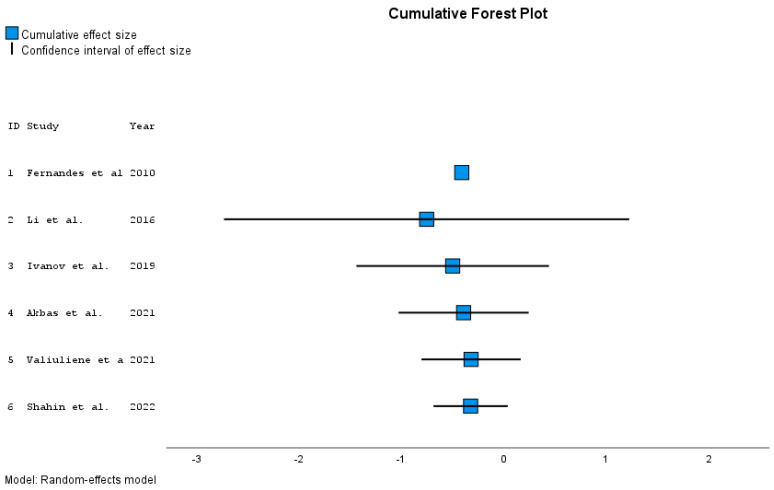
Cumulative forest plot ordered by year of publication [10,20,21,33,34,35].

Lower and upper bounds of confidence intervals for individual papers and overall effect size measured by Hedges’ g are presented in the tables with *p*-values (Table 2 and Table 3). Forest plots give summary results of meta-analyses (Figure 2 and Figure 3).

In the random-effect model, the weight w_i_ of the study depends on the observed variability (column in Table 2) according to the formula

w_i_ = 1/(SE^2^ + τ^2^).

The variability in the obtained effects for each study is due to sampling error (the error within each study SE) and the differences between the study populations (τ^2^: the variance between the studies). Values of w_i_ or relative weights define the size of squares in the forest plot (Figure 2).

The confidence interval around the variable effect (whiskers represent 95% confidence intervals) depends on τ^2^ = 0.059 and individual SE. The random effect estimates a weighted mean of the true effects of each publication. The forest plot (Figure 2) is a graphical representation of the meta-analysis, where each row represents individual study results with the effect size measure. The gray solid vertical line (x = 0) dividing the graph into two parts is the line of no effect. The dashed vertical line shows the overall effect. The blue boxes represent the individual studies, with their size reflecting the weights (estimated by inverse variance). The green diamond represents the overall effect and so does the red vertical line (described by the value about 0.328).

**Figure 4:**
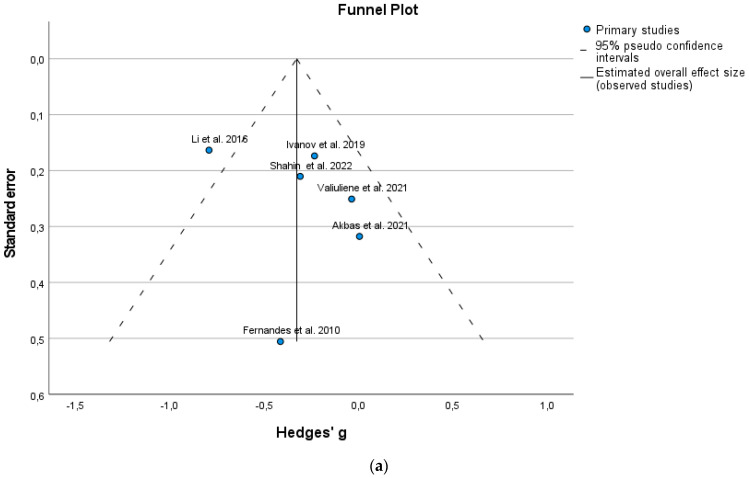

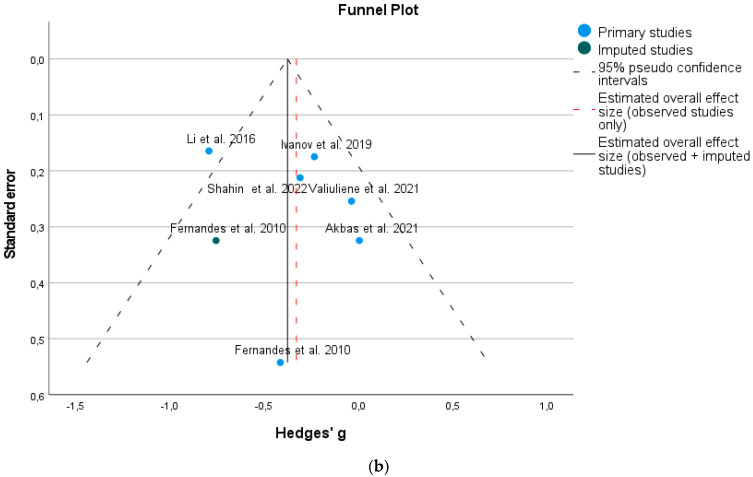
(**a**) Funnel plot, original meta-analysis. (**b**) Funnel plot, bias-corrected meta-analysis [10,20,21,33,34,35].

BDNF is a destimulant of psychotic diseases. The effect sizes (both individual and overall) have a negative sign, both for the individual and pooled effect. In the context of this meta-analysis, this represents a constructive outcome because it means that BDNF was lower after ECT. A confidence interval not reaching the gray line (zero value) suggests a significant effect, which is confirmed in Table 3. The effect measured by Hedges’ g is significant in Li et al. [10] (*p* < 0.001), while the overall effect measured by Hedges’ g is not significant (*p* = 0.069). On the other hand, only one publication imputed to the primary six studies according to the trim-and-fill procedure (Table 4) showed a significant result (*p* = 0.029). According to the Egger regression test for funnel plot asymmetry, the publication bias is not significant (Table 5; *p* = 0.185). However, from the funnel plot (Figure 4a), we can note visual asymmetry. This inconsistency can be explained by the fact that the Egger regression test has low power, especially when the number of studies is fewer than 10.

To study how the effect has changed over the years, studies are added chronologically in the analysis, and the overall effect is calculated each time. Cumulative forest plot (Figure 3) presents changes in 95% confidence intervals for effects by year of publication (corresponding Table 6 gives the detailed values). Adding publications from subsequent years increases the precision of the joint effect (corresponding whiskers representing the reduction in 95% confidence intervals).

In the funnel (triangle) plot, the studies with low precision are placed at the bottom and studies with greater precision at the top of the plot (Figure 4a,b). Funnel plots of the meta-analysis before (Figure 4a) and after applying the trim-and-fill method (Figure 4b) are given. Figure 4b shows the plot of the effect estimates from either the observed studies or both observed and imputed studies. The green circle represents the imputed result, whereas the blue circles represent the observed results. The side of the funnel plot, where the missing studies should be imputed, is chosen within the function depending on the results of Egger’s regression test. The side of imputation on the funnel plot is chosen automatically to the left (Figure 4a) on the basis of the negative Egger statistic (negative value −0.596).

The imputation of only one publication can change the result (Table 5) to obtain the effect measured by Hedges’ g −0.375 with a *p*-value smaller than the assumed significance of 0.05 (*p* = 0.029). According to Figure 4a, we can see that only one study (earliest publication by Li et al. [10]) is outside the boundary of the triangle (i.e., is not within 95% confidence intervals), but it is close to its edges.

**Figure 5:**
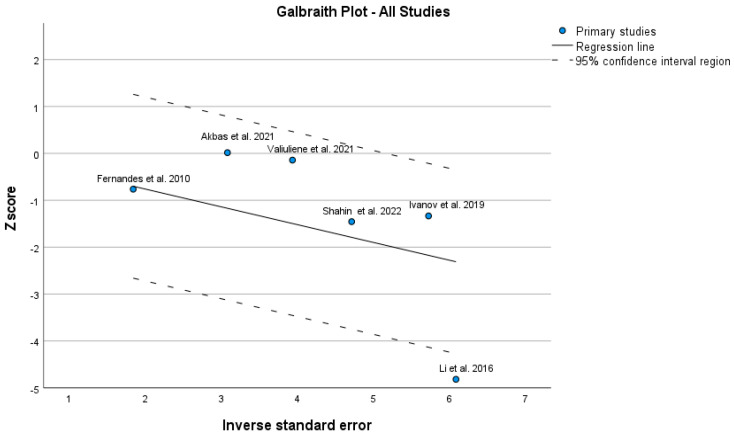
Galbraith plot [10,20,21,33,34,35].

The Galbraith plot (Figure 5) shows an unweighted regression of z-scores (each estimate is divided by its standard error) on the inverse of the standard error. It gives information about the study-specific effect sizes, their precisions and the overall effect size which can help to detect potential outliers. In the Galbraith plot, horizontal line y = 0 represents no effect. One study (again Li et al. [10]) is outside 95% confidence intervals; however, this deviation is not very apparent.

## 4. Discussion

The current meta-analysis was conducted to verify whether ECT therapy in drug-resistant schizophrenia is associated with a change in BDNF serum levels in comparison to treatment only with antipsychotic drugs. To our knowledge, to date, this is the first meta-analysis in this scope that selected exclusively patients with schizophrenia.

As is known, BDNF deficiencies play a role in the etiology of schizophrenia due to its fundamental involvement in brain function [36,37]. Several studies revealed that patients with schizophrenia have relatively lower BDNF levels compared to healthy controls [9,13,38,39,40]. Moreover, evidence showed that peripheral BDNF synthesis or release is reduced during acute episodes of schizophrenia [41,42], although it is not known whether it is a pathologic or compensatory effect. The articles included in this meta-analysis showed that, in the pre-ECT measurement, serum BDNF was decreased in drug-resistant patients with schizophrenia in comparison to healthy controls [10,20,21,33,34,35], and these differences were statistically significant.

Although there is a consensus regarding a lower BDNF level in patients with acute symptoms of schizophrenia compared to healthy persons, the changes in BDNF concentration after pharmaceutical treatment combined with ECT showed conflicting results. Recent research suggested the relative success in non-responders to pharmaceutical treatment in patients suffering from schizophrenia [43,44]. Therefore, in this meta-analysis, we expected the BDNF values to reach higher levels in the drug-resistant ECT group compared to the group that received medication only. Surprisingly, only in two studies [10,33], a statistically significant increase in the serum BDNF level after ECT treatment was observed (*p* < 0.001 and *p* < 0.027, respectively). In the case of four other studies included in this meta-analysis [20,21,34,35], ECT treatment did not cause a statistically significant change in serum BDNF levels; however, an upward trend in this matter was noticed. The estimated overall size effect revealed that ECT therapy caused a slight change in the BDNF level but without statistical significance (ES = −0.328). The obtained result may be attributed to the small sample size (overall 248 patients), various antipsychotic treatments used among patients and a short period (up to four weeks after ECT) when BDNF levels were measured. We may suspect that the BNDF levels of difficult-to-treat schizophrenia patients are more resistant to increase with treatment and may take some time after the last ECT course when an increase in the BDNF serum level will be gained. To verify this hypothesis, new studies in which BDNF levels are measured at a more extended follow-up period after completion of ECT are required. So far, Haghighi et al. [45] measured serum BDNF levels in patients with major depressive disorder (MDD), not only during ECT sessions but also at each follow-up visit after ECT up to 6 months after its completion. This study found a steady increase in BNDF up to one month after completion of ECT. However, a further increase in BDNF was less significant, and 6 months later, the BDNF level was similar to the pre-treatment levels [45,46]. Likewise, some other reports [47,48,49,50] have shown an increased level of BDNF after completion of ECT therapy in patients with MDD or in patients with bipolar disorder (BDNF levels were measured in the depressive state) [46,51,52,53].

Factors that could be responsible for the heterogeneity of studies on the BDNF level in schizophrenia have been recently identified in Ahmad et al.’s [54] review. At least some of them might influence the results of the studies on BDNF in ECT-treated individuals with schizophrenia:

1. Age, sex and weight [12,55,56];
2. Stage and/or clinical profile of disease, especially depressive symptoms [55,57,58,59,60,61,62];
3. Antipsychotic treatment [63,64];
4. Cognitive efficiency [58,59,65];
5. BDNF polymorphisms: val66met, 196g/a [66,67,68,69,70];
6. Insomnia [71];
7. Diet (i.e., curcumin, polyunsaturated fatty acids) [72,73];
8. Physical activity [74];
9. Oxidative stress [65].

Moreover, other possible variables may be considered in this context, i.e., somewhat various ECT protocols applied in studies, diverse time-frames between ECT procedure and blood sampling or inconsistent criteria of clinical improvement. Also, different sets of anaesthesiology medications used in different centers, especially ketamine, may influence the BDNF level [75]. The above-specified factors should be controlled or standardized in future research to improve the accuracy of results.

With regard to the mechanism of action, ECT induces an increase in serum BDNF levels in schizophrenia. As our meta-analysis revealed, this increase was not statistically significant; however, it may be high enough to provide beneficial effects on schizophrenia symptoms. It was found that in chronic schizophrenia, not only is there increased striatal presynaptic dopamine synthesis [76,77,78] but also a dysregulation of neurotrophic factor levels, e.g., BDNF, during brain development, which could lead to the disorganization of neuronal networks. Inadequate neurotrophic support in the adult brain may decrease its capacity to adapt to changes and increase vulnerability to neurotoxic damage [10]. Therefore, deficits in BDNF production and utilization have been implicated in the pathology of schizophrenia [79,80]. Thus, ECT therapy induces an increase in the BDNF level, which itself enhances dopamine synthesis and turnover and is involved in the maintenance of midbrain dopaminergic neurons and in the regulation of synaptic plasticity [81,82].

From the authors’ point of view, further analysis of patients’ subgroups by using the meta-regression model seems to be unfeasible, as the heterogeneity of the studies persisted. Firstly, the patients received a different number of ECT procedures (4–10 [33], 8–10 [10], 3–12 [35], 12–18 [21] up to 10–20 [34]), so the final measurement of the BDNF level (blood collected on the last day of ECT) was performed at a different time point. What is more, in most of the cases [10,20,33,34], bilateral electrode positioning was used during ECT, but due to a lack of information [21,35], unilateral electrode positioning cannot be excluded. As is known, unilateral electrode positioning during ECT is putatively associated with a lower efficacy [83] and may have an impact on BDNF production [83,84]. Also, the patients were treated with different combinations of typical (haloperidol, zuclopenthixol) and atypical antipsychotic drugs (olanzapine, risperidone, clozapine, aripiprazole, quetiapine, paliperidone). Moreover, making definitive conclusions about whether there is a relation between an increased level of BDNF after ECT and schizophrenia improvement was impossible due to a lack of complete Positive and Negative Syndrome Scale (PANSS) results or because other scales, e.g., Brief Psychiatric Rating Scale (BPRS) and Clinical Global Impression Severity scale (CGIs), [21] were used. Therefore, it is clear that new studies to evaluate BDNF levels after ECT that will take into account antipsychotic treatment, the heterogeneity of schizophrenia itself and patients’ characteristics are required. The research should be extended to a bigger group of patients and have a longer follow-up period.

## 5. Limitations

This meta-analysis has some limitations too. First, there is limited clinical literature available in this field. Identified studies include small sample sizes of patients in both groups (treated with ECT and medication or only with medication), which may have an impact on the interpretation of the results and overall obtained results. Also, the use of medications with ECT may be a confounding factor that could not be avoided. What is more, different combinations of antipsychotic medication were used during patients’ therapy, which may have a direct impact on BDNF production. Therefore, a comparison between subgroups in which particular antipsychotics were used may point to whether there are any discrepancies in this matter or if the influence of different drugs on the BDNF level is similar. In the case of our meta-analysis, this confounding effect of medication was reduced as the BDNF levels were compared by using a paired-sample test that compares each patient to themselves before and after the intervention. Another limitation is the short (four-week) follow-up assessment of the BDNF level and a lack of evaluation of this parameter in an extended period, which should be complemented with psychiatric scales, e.g., PANSS or BPRS.

Lastly, it would be worth considering an assessment of the quality of BDNF measurement in each study. The methods and precalculations in the collection and storage of the samples could likely have an impact on the obtained results. To sum up, more comprehensive research including the issues mentioned above with a larger group of patients is recommended.

## 6. Summary

Our meta-analysis showed that treatment with ECT therapy and antipsychotic medication increases serum BDNF levels in patients with drug-resistant schizophrenia compared to patients treated with medication only. Though the overall effect size measured by Hedges’ g between “before” and “after ECT” is equal to –0.328, we can conclude on the basis of these six included publications that the obtained results are not statistically significant (*p* = 0.069).

## Figures and Tables

**Table 1:** Study characteristics.

Study	Study Design	Control Group			Patients Diagnosed with Schizophrenia That Underwent ECT				Patients Diagnosed with Schizophrenia on Antipsychotic Medication Only				ECT Characteristics	BDNF Mean Level					Symptom Rating Scales before and after Intervention
		N	%F	Mean Age (years) +/− SD	N	%F	Mean Age (years) +/− SD	Duration of Illness (years) +/− SD	N	%F	Mean Age (years) +/− SD	Duration of Illness (years) +/− SD		Pre ECT	Post ECT	Med. Only Pretreatment	Med. Only Post Treatment	Control Group	
Fernandes et al. 2010 [21]	Pilot study	21	30	35.27 ± 10.34	7	30	35.79 ± 10.85	Data not available	n/a				Unilateral frontotemporal electrode placement. Charge delivered max 504 mC, current 0.9 A, frequency 30–70 Hz, pulse width 1 ms, duration max 8 s ECT session performed 3 times/week.	0.14 mg/mL	0.39 mg/mL	n/a	n/a	0.39 mg/mL	BPRS, CGI-S
Li et al. 2016 [10]	Case control study	77	44.2	40 ± 12.5	80	47.5	38.1 ± 11.1	11.3 ± 8.9	80	45	37.7 ± 12.1	11.4 ± 10.0	Bilateral frontotemporal electrode placement. Maximum charge delivered 504 mC; output current = 0.9 A; frequency between 10–70 Hz; pulse width = 0.5 ms; maximum stimulus duration = 8 s. 8–10 ECT sessions every other day.	9.7 ng/mL	11.9 ng/mL	9.8 ng/mL	11.7 ng/mL	12.4 ng/mL	PANSS
Ivanov et al. 2019 [35]	Case control study	n/a			66	50	33.28 ± 8.7	6.6 ± 5.1	32	50	33.28 ± 8.7	6.6 ± 5.1	Bilateral frontotemporal electrode placement. Maximum charge delivered 550 mC; frequency between 27–40 Hz; pulse width = 1–1.5 ms; 3–12 ECT in session	10.71 ng/mL	12.30 ng/mL	9.9 ng/mL	9.53 ng/mL	n/a	PANSS
Akbas et al. 2021 [20]	Case control study	35	0	40.51 ± 7.16	19	0	32.47 ± 9.53	7.00	35	0	35.23 ± 11.64	9.00	Bilateral temporal electrode placement. A maximum of three consecutive attempts were made to achieve adequate (25 s minimum) seizure per session.	0.320 mg/mL	0.315 mg/mL	0.141 mg/mL	0.468 mg/mL	1.478 mg/mL	PANSS
Valiuliene et al. 2021 [34]	Cohort study	19	78.9	45.53 ± 15.02	31	19	34.48 ± 11.35	Not provided	n/a				Bilateral temporal electrode placement. During the stimulation, 0.5 ms duration biphasic square impulses were applied. Impulse current strength was constant at 0.9 A. Stimulation duration ranged from 0.47 to 4.0 s at 70 Hz frequency. These parameters were adjusted according to each patient and increased gradually in succession. ECT was carried out every 2 days; the number of ECT procedures, depending on clinical progress, ranged from 10 to 20 sessions.	28.98 ng/mL	29.3 ng/mL	n/a	n/a	30.12 ng/mL	PANSS
Shahin et al. 2022 [33]	Cohort study	n/a			45	28	33.49 ± 10.14	7.43 ± 5.19	15	26.7	36.40 ± 7.18	7.60 ± 4.90	Bilateral temporal electrode placement. The baseline parameters were pulse width (0.5 milliseconds), frequency (80 Hertz), duration (1 s) and current (800 milli ampere). These parameters were adjusted according to each patient and increased gradually in successive sessions. 4–10 sessions of ECT over 4 weeks.	8.71 ng/mL	9.26 ng/mL	8.26 ng/mL	8.90 ng/mL	n/a	PANSS

BDNF—brain-derived neurotrophic factor; BPRS—the Brief Psychiatry Rating Scale; CGI-S—the Clinical Global Impression-Severity Scale; ECT—electroconvulsive therapy; %F—percent of females; Med.—patients receiving only medication; N—number of patients; PANSS—the Positive and Negative Syndrome Scale.

**Table 2:** Effect size estimates for individual studies.

ID	Study	Effect Size	Std. Error ^a^	t	Sig. (2-Tailed)	95% Confidence Interval		Weight	Weight (%)
						Lower	Upper		
1	Fernandes et al. 2010 [21]	−0.413	0.5424	−0.762	0.446	−1.477	0.650	2.832	5.7
2	Li et al. 2016 [10]	−0.792	0.1643	−4.817	<0.001	−1.114	−0.470	11.649	23.5
3	Ivanov et al. 2019 [35]	−0.233	0.1747	−1.333	0.183	−0.575	0.110	11.192	22.6
4	Akbas et al. 2021 [20]	0.005	0.3244	0.016	0.987	−0.631	0.641	6.094	12.3
5	Valiuliene et al. 2021 [34]	−0.036	0.2540	−0.143	0.887	−0.534	0.462	8.106	16.4
6	Shahin et al. 2022 [33]	−0.309	0.2121	−1.455	0.146	−0.724	0.107	9.631	19.5

^a^ Truncated Knapp–Hartung method is used for SE adjustment.

**Table 3:** Overall effect size estimates.

	Effect Size	Std. Error ^a^	t	Sig. (2-Tailed)	95% Confidence Interval		95% Prediction Interval ^b^	
					Lower	Upper	Lower	Upper
Overall	−0.328	0.1421	−2.307	0.069	−0.693	0.037	−1.108	0.453

^a^ Truncated Knapp–Hartung method is used for SE adjustment. ^b^ Based on t-distribution. Std. Error—standard error; Sig.—significance.

**Table 4:** Effect size estimates for trim-and-fill analysis.

	Number	Effect Size	Std. Error ^a^	t	Sig. (2-Tailed)	95% Confidence Interval	
						Lower	Upper
Observed	6	−0.328	0.1421	−2.307	0.069	−0.693	0.037
Observed + Imputed ^b^	7	−0.375	0.1319	−2.847	0.029	−0.698	−0.053

^a^ Truncated Knapp–Hartung method is used for SE adjustment. ^b^ Number of imputed studies: 1; Std. Error—standard error; Sig.—significance.

**Table 5:** Egger’s regression-based test ^a^.

Parameter	Coefficient	Std. Error	t	Sig. (2-Tailed)	95% Confidence Interval	
					Lower	Upper
(Intercept)	−0.596	0.3724	−1.600	0.185	−1.630	0.438
SE ^b^	1.151	1.5053	0.764	0.487	−3.029	5.330

^a^ Random-effect meta-regression with the truncated Knapp–Hartung SE adjustment. ^b^ Standard error of effect size. Std. Error—standard error; Sig.—significance.

**Table 6:** Effect size estimates for cumulative analysis.

ID	Study	Effect Size	Std. Error ^a^	t	Sig. (2-Tailed)	95% Confidence Interval		Year ^b^
						Lower	Upper	
1	Fernandes et al. 2010 [21]	−0.413	0.5424	−0.762	.	.	.	2010
2	Li et al. 2016 [10]	−0.760	0.1573	−4.831	0.130	−2.758	1,239	2016
3	Ivanov et al. 2019 [35]	−0.503	0.2219	−2.267	0.152	−1.458	0.452	2019
4	Akbas et al. 2021 [20]	−0.397	0.2019	−1.965	0.144	−1.039	0.246	2021
5	Valiuliene et al. 2021 [34]	−0.323	0.1761	−1.834	0.141	−0.812	0.166	2021
6	Shahin et al. 2022 [33]	−0.328	0.1421	−2.307	0.069	−0.693	0.037	2022

^a^ Truncated Knapp–Hartung method is used for SE adjustment; cumulative analysis based upon the variables sorted in ascending order. ^b^ Std. Error—standard error; Sig.—significance.

## Data Availability

Not applicable.

## Supplementary Materials

The following supporting information can be downloaded at: https://www.mdpi.com/article/10.3390/jcm12175728/s1, Table S1: Test of Homogeneity [24]; Table S2: Heterogeneity Measures [24].

## Abbreviations

Brain-derived neurotrophic factor (BDNF); Brief Psychiatric Rating Scale (BPRS); electroconvulsive therapy (ECT); Clinical Global Impression Severity scale (CGIs); major depressive disorder (MDD); Positive and Negative Syndrome Scale (PANSS).

## References

[1] Keshri N., Nandeesha H. (2023). Dysregulation of Synaptic Plasticity Markers in Schizophrenia. Indian J. Clin. Biochem..

[2] Patlola S.R., Donohoe G., McKernan D.P. (2023). The relationship between inflammatory biomarkers and cognitive dysfunction in patients with schizophrenia: A systematic review and meta-analysis. Prog. Neuro-Psychopharmacol. Biol. Psychiatry.

[3] Zoupa E., Bogiatzidou O., Siokas V., Liampas I., Tzeferakos G., Mavreas V., Stylianidis S., Dardiotis E. (2022). Cognitive Rehabilitation in Schizophrenia-Associated Cognitive Impairment: A Review. Neurol. Int..

[4] Ashe P.C., Berry M.D., Boulton A.A. (2001). Schizophrenia, a neurodegenerative disorder with neurodevelopmental antecedents. Prog. Neuro-Psychopharmacol. Biol. Psychiatry.

[5] Bathina S., Das U.N. (2015). Brain-derived neurotrophic factor and its clinical implications. Arch. Med. Sci..

[6] Scalzo P., Kümmer A., Bretas T.L., Cardoso F., Teixeira A.L. (2010). Serum levels of brain-derived neurotrophic factor correlate with motor impairment in Parkinson’s disease. J. Neurol..

[7] Ventriglia M., Zanardini R., Bonomini C., Zanetti O., Volpe D., Pasqualetti P., Gennarelli M., Bocchio-Chiavetto L. (2013). Serum brain-derived neurotrophic factor levels in different neurological diseases. BioMed Res. Int..

[8] Zheng F., Zhou X., Moon C., Wang H. (2012). Regulation of brain-derived neurotrophic factor expression in neurons. Int. J. Physiol. Pathophysiol. Pharmacol..

[9] Green M.J., Matheson S.L., Shepherd A., Weickert C.S., Carr V.J. (2011). Brain-derived neurotrophic factor levels in schizophrenia: A systematic review with meta-analysis. Mol. Psychiatry.

[10] Li J., Ye F., Xiao W., Tang X., Sha W., Zhang X., Wang J. (2016). Increased serum brain-derived neurotrophic factor levels following electroconvulsive therapy or antipsychotic treatment in patients with schizophrenia. Eur. Psychiatry.

[11] Şimşek Ş., Gençoğlan S., Yüksel T., Kaplan İ., Aktaş H. (2015). Lower Brain-Derived Neurotropic Factor Levels in Untreated Adolescents with First-Episode Psychosis. J. Clin. Psychopharmacol..

[12] Weickert C.S., Lee C.H., Lenroot R.K., Bruggemann J., Galletly C., Liu D., Balzan R., Pillai A., Buckley P., Weickert T.W. (2019). Increased plasma Brain-Derived Neurotrophic Factor (BDNF) levels in females with schizophrenia. Schizophr. Res..

[13] Ray M.T., Shannon T., Weickert C., Webster M.J. (2014). Decreased BDNF and TrkB mRNA expression in multiple cortical areas of patients with schizophrenia and mood disorders. Transl. Psychiatry.

[14] Wong J., Hyde T.M., Cassano H.L., Deep-Soboslay A., Kleinman J.E., Weickert C.S. (2010). Promoter specific alterations of brain-derived neurotrophic factor mRNA in schizophrenia. Neuroscience.

[15] Fernandes B.S., Steiner J., Berk M., Molendijk M.L., Gonzalez-Pinto A., Turck C.W., Nardin P., Gonçalves C.A. (2015). Peripheral brain-derived neurotrophic factor in schizophrenia and the role of antipsychotics: Meta-analysis and implications. Mol. Psychiatry.

[16] Gama C.S., Andreazza A.C., Kunz M., Berk M., Belmonte-de-Abreu P.S., Kapczinski F. (2007). Serum levels of brain-derived neurotrophic factor in patients with schizophrenia and bipolar disorder. Neurosci. Lett..

[17] Reis H.J., Nicolato R., Barbosa I.G., Teixeira do Prado P.H., Romano-Silva M.A., Teixeira A.L. (2008). Increased serum levels of brain-derived neurotrophic factor in chronic institutionalized patients with schizophrenia. Neurosci. Lett..

[18] Pedrini M., Chendo I., Grande I., Lobato M.I., Belmonte-de-Abreu P.S., Lersch C., Walz J., Kauer-Sant’anna M., Kapczinski F., Gama C.S. (2011). Serum brain-derived neurotrophic factor and clozapine daily dose in patients with schizophrenia: A positive correlation. Neurosci. Lett..

[19] Leucht S. (2014). Measurements of response, remission, and recovery in schizophrenia and examples for their clinical application. J. Clin. Psychiatry.

[20] Akbas I., Balaban O.D. (2022). Changes in serum levels of brain-derived neurotrophic factor with electroconvulsive therapy and pharmacotherapy and its clinical correlates in male schizophrenia patients. Acta Neuropsychiatr..

[21] Fernandes B.S., Massuda R., Torres M., Camargo D., Fries G.R., Gama C.S., Belmonte-de-Abreu P.S., Kapczinski F., Lobato M.I. (2010). Improvement of schizophrenia with electroconvulsive therapy and serum brain-derived neurotrophic factor levels: Lack of association in a pilot study. Psychiatry Clin. Neurosci..

[22] Page M.J., McKenzie J.E., Bossuyt P.M., Boutron I., Hoffmann T.C., Mulrow C.D., Shamseer L., Tetzlaff J.M., Akl E.A., Brennan S.E. (2021). The PRISMA 2020 statement: An updated guideline for reporting systematic reviews. BMJ.

[23] Hedges L. (1981). Distribution Theory for Glass’s Estimator of Effect Size and Related Estimators. J. Edu. Stat..

[24] Hedges L.V., Olkin I. (1985). Statistical Methods for Meta-Analysis.

[25] Knapp G., Hartung J. (2003). Improved tests for a random effects meta-regression with a single covariate. Stat. Med..

[26] IntHout J., Ioannidis J.P., Borm G.F. (2014). The Hartung-Knapp-Sidik-Jonkman method for random effects meta-analysis is straightforward and considerably outperforms the standard DerSimonian-Laird method. BMC Med. Res. Methodol..

[27] Röver C., Knapp G., Friede T. (2015). Hartung-Knapp-Sidik-Jonkman approach and its modification for random-effects meta-analysis with few studies. BMC Med. Res. Meth..

[28] Simes R.J. (1987). Confronting publication bias: A cohort design for meta-analysis. Stat. Med..

[29] Egger M., Davey Smith G., Schneider M., Minder C. (1997). Bias in meta-analysis detected by a simple, graphical test. BMJ.

[30] Sterne J.A.C., Egger M., Rothstein H.R., Sutton A.J., Borenstein M. (2005). Regression methods to detect publication and other bias in meta-analysis. Publication Bias in Meta-Analysis: Prevention, Assessment, and Adjustments.

[31] Duval S., Tweedie R. (2000). Trim and fill: A simple funnel-plot-based method of testing and adjusting for publication bias in meta-analysis. Biometrics.

[32] Linyu S., Lifeng L. (2019). The trim-and-fill method for publication bias: Practical guidelines and recommendations based on a large database of meta-analyses. Medicine.

[33] Shahin O., Gohar S.M., Ibrahim W., El-Makawi S.M., Fakher W., Taher D.B., Abdel Samie M., Khalil M.A., Saleh A.A. (2022). Brain-Derived neurotrophic factor (BDNF) plasma level increases in patients with resistant schizophrenia treated with electroconvulsive therapy (ECT). Int. J. Psychiatry Clin. Pract..

[34] Valiuliene G., Valiulis V., Dapsys K., Vitkeviciene A., Gerulskis G., Navakauskiene R., Germanavicius A. (2021). Brain stimulation effects on serum BDNF, VEGF, and TNFα in treatment-resistant psychiatric disorders. Eur. J. Neurosci..

[35] Ivanov M.V., Zubov D.S. (2019). Electroconvulsive therapy in treatment of resistant schizophrenia: Biological markers of efficacy and safety. Zhurnal Nevrol. I Psikhiatrii Im. S.S. Korsakova.

[36] Lu B., Nagappan G., Lu Y. (2014). BDNF and synaptic plasticity, cognitive function, and dysfunction. Handbook of Experimental Pharmacology.

[37] Nagahara A.H., Tuszynski M.H. (2011). Potential therapeutic uses of BDNF in neurological and psychiatric disorders. Nat. Rev. Drug Discov..

[38] Akyol E.S., Albayrak Y., Beyazyüz M., Aksoy N., Kuloglu M., Hashimoto K. (2015). Decreased serum levels of brain-derived neurotrophic factor in schizophrenic patients with deficit syndrome. Neuropsychiatr. Dis. Treat..

[39] Islam F., Mulsant B.H., Voineskos A.N., Rajji T.K. (2017). Brain-Derived Neurotrophic Factor Expression in Individuals with Schizophrenia and Healthy Aging: Testing the Accelerated Aging Hypothesis of Schizophrenia. Curr. Psychiatry Rep..

[40] Koeva Y.A., Sivkov S.T., Akabaliev V.H. (2014). Brain-derived neurotrophic factor and its serum levels in schizophrenic patients. Folia Medica.

[41] Peng S., Li W., Lv L., Zhang Z., Zhan X. (2018). BDNF as a biomarker in diagnosis and evaluation of treatment for schizophrenia and depression. Discov. Med..

[42] Zakharyan R., Boyajyan A. (2014). Brain-derived neurotrophic factor blood levels are decreased in schizophrenia patients and associate with rs6265 genotypes. Clin. Biochem..

[43] Chan C.Y.W., Abdin E., Seow E., Subramaniam M., Liu J., Peh C.X., Tor P.C. (2019). Clinical effectiveness and speed of response of electroconvulsive therapy in treatment-resistant schizophrenia. Psychiatry Clin. Neurosci..

[44] Petrides G., Malur C., Braga R.J., Bailine S.H., Schooler N.R., Malhotra A.K., Kane J.M., Sanghani S., Goldberg T.E., John M. (2015). Electroconvulsive therapy augmentation in clozapine-resistant schizophrenia: A prospective, randomized study. Am. J. Psychiatry.

[45] Haghighi M., Salehi I., Erfani P., Jahangard L., Bajoghli H., Holsboer-Trachsler E., Brand S. (2013). Additional ECT increases BDNF-levels in patients suffering from major depressive disorders compared to patients treated with citalopram only. J. Psychiatr. Res..

[46] Luan S., Zhou B., Wu Q., Wan H., Li H. (2020). Brain-derived neurotrophic factor blood levels after electroconvulsive therapy in patients with major depressive disorder: A systematic review and meta-analysis. Asian J. Psychiatry.

[47] Brunoni A.R., Baeken C., Machado-Vieira R., Gattaz W.F., Vanderhasselt M.A. (2014). BDNF blood levels after electroconvulsive therapy in patients with mood disorders: A systematic review and meta-analysis. World J. Biol. Psychiatry.

[48] Pelosof R., Santos L.A.D., Farhat L.C., Gattaz W.F., Talib L., Brunoni A.R. (2023). BDNF blood levels after electroconvulsive therapy in patients with mood disorders: An updated systematic review and meta-analysis. World J. Biol. Psychiatry.

[49] Polyakova M., Schroeter M.L., Elzinga B.M., Holiga S., Schoenknecht P., de Kloet E.R., Molendijk M.L. (2015). Brain-Derived Neurotrophic Factor and Antidepressive Effect of Electroconvulsive Therapy: Systematic Review and Meta-Analyses of the Preclinical and Clinical Literature. PLoS ONE.

[50] Salehi I., Hosseini S.M., Haghighi M., Jahangard L., Bajoghli H., Gerber M., Pühse U., Holsboer-Trachsler E., Brand S. (2016). Electroconvulsive therapy (ECT) and aerobic exercise training (AET) increased plasma BDNF and ameliorated depressive symptoms in patients suffering from major depressive disorder. J. Psychiatr. Res..

[51] Fernandes B., Molendijk M.L., Köhler C.A., Soares J.C., Leite C.M., Machado-Vieira R., Ribeiro T.L., Silva J.C., Sales P.M., Quevedo J. (2015). Peripheral brain-derived neurotrophic factor (BDNF) as a biomarker in bipolar disorder: A meta-analysis of 52 studies. BMC Med..

[52] Grønli O., Stensland G.Ø., Wynn R., Olstad R. (2009). Neurotrophic factors in serum following ECT: A pilot study. World J. Biol. Psychiatry.

[53] Okamoto T., Yoshimura R., Ikenouchi-Sugita A., Hori H., Umene-Nakano W., Inoue Y., Ueda N., Nakamura J. (2008). Efficacy of electroconvulsive therapy is associated with changing blood levels of homovanillic acid and brain-derived neurotrophic factor (BDNF) in refractory depressed patients: A pilot study. Prog. Neuro-Psychopharmacol. Biol. Psychiatry.

[54] Ahmad R., Azman K.F., Yahaya R., Shafin N., Omar N., Ahmad A.H., Zakaria R., Wijaya A., Othman Z. (2023). Brain-derived neurotrophic factor (BDNF) in schizophrenia research: A quantitative review and future directions. AIMS Neurosci..

[55] Atake K., Nakamura T., Ueda N., Hori H., Katsuki A., Yoshimura R. (2018). The Impact of Aging, Psychotic Symptoms, Medication, and Brain-Derived Neurotrophic Factor on Cognitive Impairment in Japanese Chronic Schizophrenia Patients. Front. Psychiatry.

[56] Yang F., Wang K., Du X., Deng H., Wu H.E., Yin G., Ning Y., Huang X., Teixeira A.L., de Quevedo J. (2019). Sex difference in the association of body mass index and BDNF levels in Chinese patients with chronic schizophrenia. Psychopharmacology.

[57] Binford S.S., Hubbard E.M., Flowers E., Miller B.L., Leutwyler H. (2018). Serum BDNF Is Positively Associated with Negative Symptoms in Older Adults with Schizophrenia. Biol. Res. Nurs..

[58] Heitz U., Papmeyer M., Studerus E., Egloff L., Ittig S., Andreou C., Vogel T., Borgwardt S., Graf M., Eckert A. (2019). Plasma and serum brain-derived neurotrophic factor (BDNF) levels and their association with neurocognition in at-risk mental state, first episode psychosis and chronic schizophrenia patients. World J. Biol. Psychiatry.

[59] Tang X., Zhou C., Gao J., Duan W., Yu M., Xiao W., Zhang X., Dong H., Wang X., Zhang X. (2019). Serum BDNF and GDNF in Chinese male patients with deficit schizophrenia and their relationships with neurocognitive dysfunction. BMC Psychiatry.

[60] Fang X., Chen Y., Wang Y., Ren J., Zhang C. (2019). Depressive symptoms in schizophrenia patients: A possible relationship between SIRT1 and BDNF. Prog. Neuro-Psychopharmacol. Biol. Psychiatry.

[61] Li S., Lu C., Kang L., Li Q., Chen H., Zhang H., Tang Z., Lin Y., Bai M., Xiong P. (2023). Study on correlations of BDNF, PI3K, AKT and CREB levels with depressive emotion and impulsive behaviors in drug-naïve patients with first-episode schizophrenia. BMC Psychiatry.

[62] Manchia M., Isayeva U., Collu R., Primavera D., Deriu L., Caboni E., Iaselli M.N., Sundas D., Tusconi M., Pinna F. (2022). Converging Evidence Points to BDNF as Biomarker of Depressive Symptoms in Schizophrenia-Spectrum Disorders. Brain Sci..

[63] Huang T.L. (2013). Effects of antipsychotics on the BDNF in schizophrenia. Curr. Med. Chem..

[64] Wu R.Q., Lin C.G., Zhang W., Lin X.D., Chen X.S., Chen C., Zhang L.J., Huang Z.Y., Chen G.D., Xu D.L. (2018). Effects of Risperidone and Paliperidone on Brain-Derived Neurotrophic Factor and N400 in First-Episode Schizophrenia. Chin. Med. J..

[65] Wei C., Sun Y., Chen N., Chen S., Xiu M., Zhang X. (2020). Interaction of oxidative stress and BDNF on executive dysfunction in patients with chronic schizophrenia. Psychoneuroendocrinology.

[66] Xia H., Zhang G., Du X., Zhang Y., Yin G., Dai J., He M.X., Soares J.C., Li X., Zhang X. (2018). Suicide attempt, clinical correlates, and BDNF Val66Met polymorphism in chronic patients with schizophrenia. Neuropsychology.

[67] Skibinska M., Groszewska A., Kapelski P., Rajewska-Rager A., Pawlak J., Dmitrzak-Weglarz M., Szczepankiewicz A., Twarowska-Hauser J. (2018). Val66Met functional polymorphism and serum protein level of brain-derived neurotrophic factor (BDNF) in acute episode of schizophrenia and depression. Pharmacol. Rep..

[68] Huang E., Hettige N.C., Zai G., Tomasi J., Huang J., Zai C.C., Pivac N., Nikolac Perkovic M., Tiwari A.K., Kennedy J.L. (2019). BDNF Val66Met polymorphism and clinical response to antipsychotic treatment in schizophrenia and schizoaffective disorder patients: A meta-analysis. Pharmacogenomics J..

[69] Kim E.J., Kim Y.K. (2018). 196G/A of the Brain-derived neurotrophic factor gene polymorphisms predicts suicidal behavior in schizophrenia patients. Psychiatry Investig..

[70] Schweiger J.I., Bilek E., Schäfer A., Braun U., Moessnang C., Harneit A., Post P., Otto K., Romanczuk-Seiferth N., Erk S. (2019). Effects of BDNF Val^66^Met genotype and schizophrenia familial risk on a neural functional network for cognitive control in humans. Neuropsychopharmacology.

[71] Schmitt K., Holsboer-Trachsler E., Eckert A. (2016). BDNF in sleep, insomnia, and sleep deprivation. Ann. Med..

[72] Wynn J.K., Green M.F., Hellemann G., Karunaratne K., Davis M.C., Marder S.R. (2018). The effects of curcumin on brain-derived neurotrophic factor and cognition in schizophrenia: A randomized controlled study. Schizophr. Res..

[73] Pawełczyk T., Grancow-Grabka M., Trafalska E., Szemraj J., Żurner N., Pawełczyk A. (2019). An increase in plasma brain derived neurotrophic factor levels is related to n-3 polyunsaturated fatty acid efficacy in first episode schizophrenia: Secondary outcome analysis of the OFFER randomized clinical trial. Psychopharmacology.

[74] Gökçe E., Güneş E., Nalçaci E. (2019). Effect of Exercise on Major Depressive Disorder and Schizophrenia: A BDNF Focused Approach. Noro Psikiyatr. Ars..

[75] Meshkat S., Alnefeesi Y., Jawad M.Y.D., Di Vincenzo J.B., Rodrigues N., Ceban F., Mw Lui L., McIntyre R.S., Rosenblat J.D. (2022). Brain-Derived Neurotrophic Factor (BDNF) as a biomarker of treatment response in patients with Treatment Resistant Depression (TRD): A systematic review & meta-analysis. Psychiatry Res..

[76] Faden J., Citrome L. (2023). Schizophrenia: One Name, Many Different Manifestations. Med. Clin. N. Am..

[77] Howes O.D., Murray R.M. (2014). Schizophrenia: An integrated sociodevelopmental-cognitive model. Lancet.

[78] Xu H., Yang F. (2022). The interplay of dopamine metabolism abnormalities and mitochondrial defects in the pathogenesis of schizophrenia. Transl. Psychiatry.

[79] Nieto R.R., Carrasco A., Corral S., Castillo R., Gaspar P.A., Bustamante M.L., Silva H. (2021). BDNF as a Biomarker of Cognition in Schizophrenia/Psychosis: An Updated Review. Front. Psychiatry.

[80] Zhang H.C., Du Y., Chen L., Yuan Z.Q., Cheng Y. (2023). MicroRNA schizophrenia: Etiology, biomarkers and therapeutic targets. Neurosci. Biobehav. Rev..

[81] Nucifora F.C., Woznica E., Lee B.J., Cascella N., Sawa A. (2019). Treatment resistant schizophrenia: Clinical, biological, and therapeutic perspectives. Neurobiol. Dis..

[82] Rosenquist P.B., Miller B., Pillai A. (2014). The antipsychotic effects of ECT: A review of possible mechanisms. J. ECT.

[83] Kellner C.H., Knapp R., Husain M.M., Rasmussen K., Sampson S., Cullum M., McClintock S.M., Tobias K.G., Martino C., Mueller M. (2010). Bifrontal, bitemporal and right unilateral electrode placement in ECT: Randomised trial. Br. J. Psychiatry.

[84] Swartz C.M., Nelson A.I. (2005). Rational electroconvulsive therapy electrode placement. Psychiatry.

