# Supplementary material for: The Influence of Electroconvulsive Therapy (ECT) on Brain-Derived Neurotrophic Factor (BDNF) Plasma Level in Patients with Schizophrenia—A Systematic Review and Meta-Analysis
Source: J Clin Med. 2023 Sep 2;12(17):5728. doi: 10.3390/jcm12175728 (PMC10488522; doi:10.3390/jcm12175728)
Supplement: Supplementary file 1 [file jcm-12-05728-s001.zip › jcm-2553958-supplementary.pdf]

Table S1. Test of Homogeneity [24]

	Chi-square (Q statistic)	Degrees of freedom	Significance.
Overall	10,342	5	0,066

Table S2. Heterogeneity Measures [24]

	Measure	Value
Overall	Tau-squared	0,059
	H-squared	2,096
	I-squared (%)	52,3
